# Hemorrhagic Fever Successfully Treated without Quinine

**Published:** 1881-02-20

**Authors:** E. H. M. Parham


### HEMORRHAGIC FEVER SUCCESSFULLY TREATED WITHOUT QUININE.

Twelve or thirteen years since, my attention was directed to the first case of swamp or hemorrhagic fever that I had ever seen. From that time to the present, such cases have become more frequent annually; until it ceases to be uncommon to see many every autumn, and in some localities attended with great fatality. What the treatment was in the fatal cases, I do not know, but suppose that quinine was used in most or all of them.

The first case I saw, which was a violent one, I tried quinine in sufficient quantities during the morning remissions, but soon found that it aggravated every symptom, and I therefore abandoned it, in time to relieve my patient. Having had many cases of that dreadful form of fever to treat without losing but one, and that one having been under treatment for organic heart disease six months preceding his attack. I therefore feel that it is a duty which I owe to the profession to give a brief account of my simple but efficient plan of treatment, and in doing so I can think of no better way than to report a case in point:

On September 19th, Mr. A. J. C. sent to me for a cathartic and quinine sufficient to stop his chills, stating that he had a very severe one that day. I sent him a purgative dose of calomel, comp. ext. of colocynth and ipecac, and 20 grs. of quinine.

On the 20th, I was sent for in haste to see him. On my arrival, he stated that the cathartic had acted mildly but sufficiently, and as soon as he had taken a few doses of quinine that he commenced vomiting and blood flowed freely from the kidneys. I found his pulse 120, corded and feeble, tongue coated, thirst intense, and his stomach ejecting a bluish-colored substance, skin jaundiced, and nausea continuous. Prescribed calomel 1 gr., Dover's powder 4 grs. every four hours, alternated with 10 drop doses of fluid extract of ergot to be given in buchu leaf tea.

On the morning of the 21st, I visited the patient again and found no material change in his condition; ordered the prescription continued, except that the ergot to be substituted with 10 drop doses of the tincture of the muriate of iron, to be given in the same way, as the ergot was very offensive to his taste.

On the evening of the same day, I visited the patient again; no alteration in his condition, except that his skin was more deeply jaundiced, and he was passing blood profusely from his bowels as well as his kidneys. Ordered a large blister over the region of the liver and stomach, and prescription continued, and I remained with the patient until the blister had drawn, and on examination I found that it had the appearance of a blood blister, but on evacuating the contents, I found it to be bilious matter of a deep yellow color.

In the evening of the 22d, I visited my patient again, and found him very feeble; circulation increased in volume and less frequent, dejections small and thick, but the color of blood; vomiting less frequent, and his gums slightly touched with mercury. Ordered tincture of iron in buchu leaf tea, three time per diem.

On the 23d, visited my patient again and found him without fever, some appetite, no nausea, and no hemorrhage, and comfortable.

On the 24th, I found him much improved in every respect; and on the 25th discharged him.

The case given above is one among many, about the same treatment and same result. I offer no comment, as the motto of the SOUTHERN MEDICAL RECORD is "*Quicquid Præcipies Esto Brevis.*" But I will say this, I believe that hemorrhagic fever, at this time, demands more serious attention from learned physicians than any other disease with which the Southwestern States are troubled.

If this should be found worthy of publication, I hope that members of our profession will give it a trial and report the result.

E. H. M. PARHAM, M. D.

